# Impact of social support on the outcomes of upper limb occupational injuries

**DOI:** 10.1192/j.eurpsy.2025.1591

**Published:** 2025-08-26

**Authors:** A. Hrairi, N. Rmadi, M. Hajji, N. Kotti, A. Haddar, M. Hajjaji, K. Jmal Hammami

**Affiliations:** 1 Occupational medicine department, Hedi chaker university hospital, University of Sfax; 2 Family medicine department, University of Sfax, Sfax, Tunisia

## Abstract

**Introduction:**

Upper limb occupational injuries constitute an important health problem affecting workers in their most productive years. The professional environment influences the outcomes of these injuries. The impact of social support among this vulnerable population may explain the difference in terms of outcomes of occupational injury.

**Objectives:**

Evaluating the impact of social support in upper limb occupational injuries’ outcomes.

**Methods:**

A cross-sectional analysis was conducted during 9 months among workers victims of upper limb occupational injuries consulting for an Impairment Rating Evaluation. Socio-professional data and the accident’ outcomes were collected. Social support was evaluated by the Social Support Scale. The pain was evaluated by a Visual Analogue Scale. Anxiety and depression were evaluated by the Arabic version of Hospital Anxiety and Depression scale. Unsuccessful return to work comprises all situations other than a satisfactory return to the same position held before the accident.

**Results:**

Out of 90 injured workers, 78.9% were male. Hand and wrist injuries represented 63% of injured sites. The mean age was 43.10± 9.87 years. The mean pain scale was 5.75±2.78. The mean length of absence was 180.73±245.14 days. A proportion of 12.2 % had low social support. Unsuccessful return to work was found among 37.8% of participants. The prevalence of anxiety and depression were 31.1% and 20% respectively. Sleep disorders were mentionned by 56.7% of subjects. Low social support was associated with unsuccessful return to work (p=0.000), negative outlook of the professional future (p=0.000) and depression (p=0.002). No association was found with pain, length of absence and sleep disorders.

**Conclusions:**

Social support may influence the outcomes of upper limbs occupational injuries. This finding highlights the need for further examination of social factors among this vulnerable population.

**Disclosure of Interest:**

None Declared

